# Legg-Calve-Perthes disease in an 8-year old girl with Acrodysostosis type 1 on growth hormone therapy: case report

**DOI:** 10.1186/s13633-020-00085-3

**Published:** 2020-08-07

**Authors:** Whei Ying Lim, Emily L. Germain-Lee, Nancy S. Dunbar

**Affiliations:** 1 Division of Pediatric Endocrinology, Connecticut Children’s, 505 Farmington Avenue, Farmington, CT 06032 USA; 2 University of Connecticut School of Medicine, 200 Academic Way, Farmington, CT 06032 USA

**Keywords:** Acrodysostosis, Growth hormone, Legg-Calve-Perthes disease, Pseudohypoparathyroidism

## Abstract

**Background:**

Acrodyostosis type 1 (ACRDYS1) is a rare skeletal dysplasia, and sometimes it can be misdiagnosed as pseudohypoparathyroidism type 1A (PHP1A), a subtype of Albright hereditary osteodystrophy (AHO), due to overlapping features. Growth hormone releasing hormone (GHRH) resistance with severe short stature is common in both ACRDYS1 and PHP1A (Emily L. Germain-Lee, et al. J Clin Endocrinol Metab, 88:4059–4069, 2003). Whereas growth hormone (GH) treatment has been studied in patients with PHP1a, the same is not true for the rarer ACRDYS1. Here in we report an adverse orthopedic outcome in a patient with ACRDYS1 with severe short stature treated with growth hormone. Our experience could have implications for the treatment of other patients with this disorder.

**Case presentation:**

We report a case of Legg-Calve-Perthes Disease (LCPD) in an 8-year old female with ACRDYS1 treated with GH. She initially presented with marked short stature (height Z-score − 3.46) with a low normal insulin like growth factor-1 (IGF1) level, and had biochemical evidence of thyrotropin and parathyroid hormone resistance. GH therapy was initiated at 0.35 mg/kg/week leading to increased growth velocity. After 7 months on GH, she developed right knee pain. Radiographic images revealed flattening of her right femoral head consistent with LCPD. GH was discontinued. Six weeks later, radiographs revealed further collapse of the entire femoral head. Her lesion stabilized after 8 months with conservative management and she never resumed GH. Her final adult height is 4′2″ (128 cm).

**Conclusion:**

Patients with ACRDYS1 on GH therapy may be at increased risk of LCPD. This has not been reported in patients with PHP1A treated with GH. Clinicians and families need to be aware of this potential complication when counseling about GH treatment.

## Background

Acrodysostosis (ACRDYS) is a rare skeletal dysplasia with skeletal abnormalities and multi-hormonal resistance similar to PHP1A, but caused by mutations downstream of the genes involved in PHP1A. Whereas PHP1A results from mutations of the gene encoding the Gα-stimulatory subunit (*GNAS*), ACRDYS is caused by mutations involving the genes for downstream effector proteins of *GNAS* in the cyclic AMP (cAMP)/ protein kinase A (PKA) pathway. In ACRDYS1, the protein kinase A regulatory subunit 1A (*PRKAR1A*) is mutated and in ACRDYS2 the gene coding for phosphodiestarase type 4D (*PDE4D*) is mutated. *PRKAR1A* encodes the cAMP-dependent regulatory subunit of protein kinase A. The mutated subunit impairs the protein kinase A response to cAMP accounting for the hormonal resistance and skeletal abnormalities in ACRDYS1. *PDE4D* is also involved in the cAMP signaling pathway, encoding a class-IV cAMP-specific phosphodiesterase, regulating cAMP concentrations and leading to ACRDYS2 [1]. The two subtypes of ACRDYS are both characterized by similar skeletal features (short stature, facial dysostosis, and brachydactyly with cone-shaped epiphyses) and variable multi-hormone resistance. Patients with ACRDYS1 tend to have less facial dysostosis, and normal intelligence with multi-hormone resistance. Patients with ACRDYS2 tend to have more characteristic facial dysostosis and intellectual disabilities but have only subtle or absent hormonal resistance [1].

The G-protein coupled hormones which are commonly affected in ACRDYS1 include parathyroid hormone (PTH), thyroid-stimulating hormone (TSH), growth hormone-releasing hormone (GHRH), luteinizing hormone (LH), and follicle-stimulating hormone (FSH). Accepted standard therapy involves thyroid hormone replacement and calcitriol to normalize thyroid and calcium metabolism. Growth hormone (GH) therapy is an accepted treatment for PHP1A patients with short stature and GH deficiency [2] but efficacy of GH therapy is unknown in ACRDYS1. We report a case of ACRDYS1 in an 8-year-old female with severe short stature treated empirically with GH which was complicated by LCPD.

## Case presentation

An 8-year-old female was referred to pediatric endocrinology for short stature. She was born at 38 weeks and small for gestational age (SGA) - birth weight of 4 pounds 8 oz (2.04 kg), and birth length of 16 in. (40.6 cm). Her father was from Mexico and her mother was from Puerto Rico and their heights are 5′9″ (175.3 cm) and 5′5″ (165.1 cm) respectively giving her a mid-parental height of 5′4.5″ (163.8 cm). She had normal development, without any significant medical problems.

**Fig. 1:**
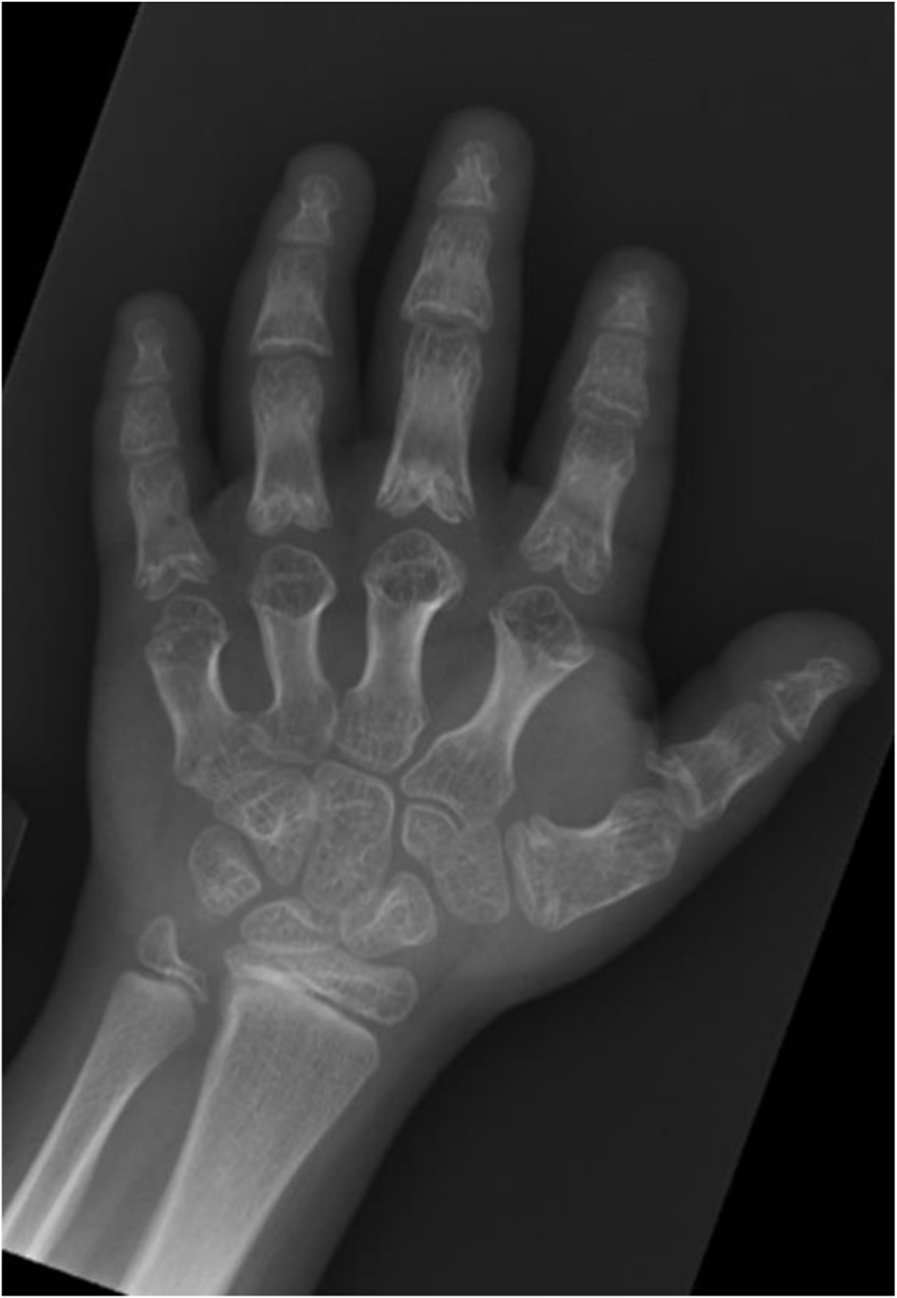
Brachydactyly and cone shaped epiphyses seen in patient which is characteristic of ACRDYS

Physical examination demonstrated severe short stature (height Z-score − 3.46), midface hypoplasia, and severe brachydactyly of her hands and feet. Laboratory analysis showed mildly elevated TSH with normal free thyroxine, mildly low calcium with elevated PTH and low normal IGF-1 level (Table 1). Radiographs revealed severe brachymetatarsia, brachymetacarpia, and brachydactyly with cone-shaped epiphyses in the proximal phalanges (Fig. 1). Genetic testing revealed a mutation of the *PRKAR1A* gene, specifically R368X (CGA > TGA). To address her TSH and PTH resistance, she was started on levothyroxine and calcitriol. We postulated that her severe short stature was from a combination of GHRH resistance and being born SGA. GH therapy was initiated at 0.35 mg/kg/week – a mid-range dose for children with SGA. Three months later, her serum IGF-1 Z-score increased from − 1.2 to 2.1 at an effective GH dose of 0.33 mg/kg/week.

**Table 1:** Laboratory analysis upon initial presentation at age 8 years

	Reference range	Results
TSH	0.6–5.5 μU/ml	10.6
Free T4	0.9–1.4 ng/dl	1.0
PTH	11–74 pg/ml	149
Calcium	8.9–10.4 mg/dl	8.7
Phosphorus	3–6 mg/dl	4.8
IGF-1	76–424 ng/ml	91 (z score − 1.2)
IGF-BP3	1.6–6.5 mg/dl	3.0

**Fig. 2:**
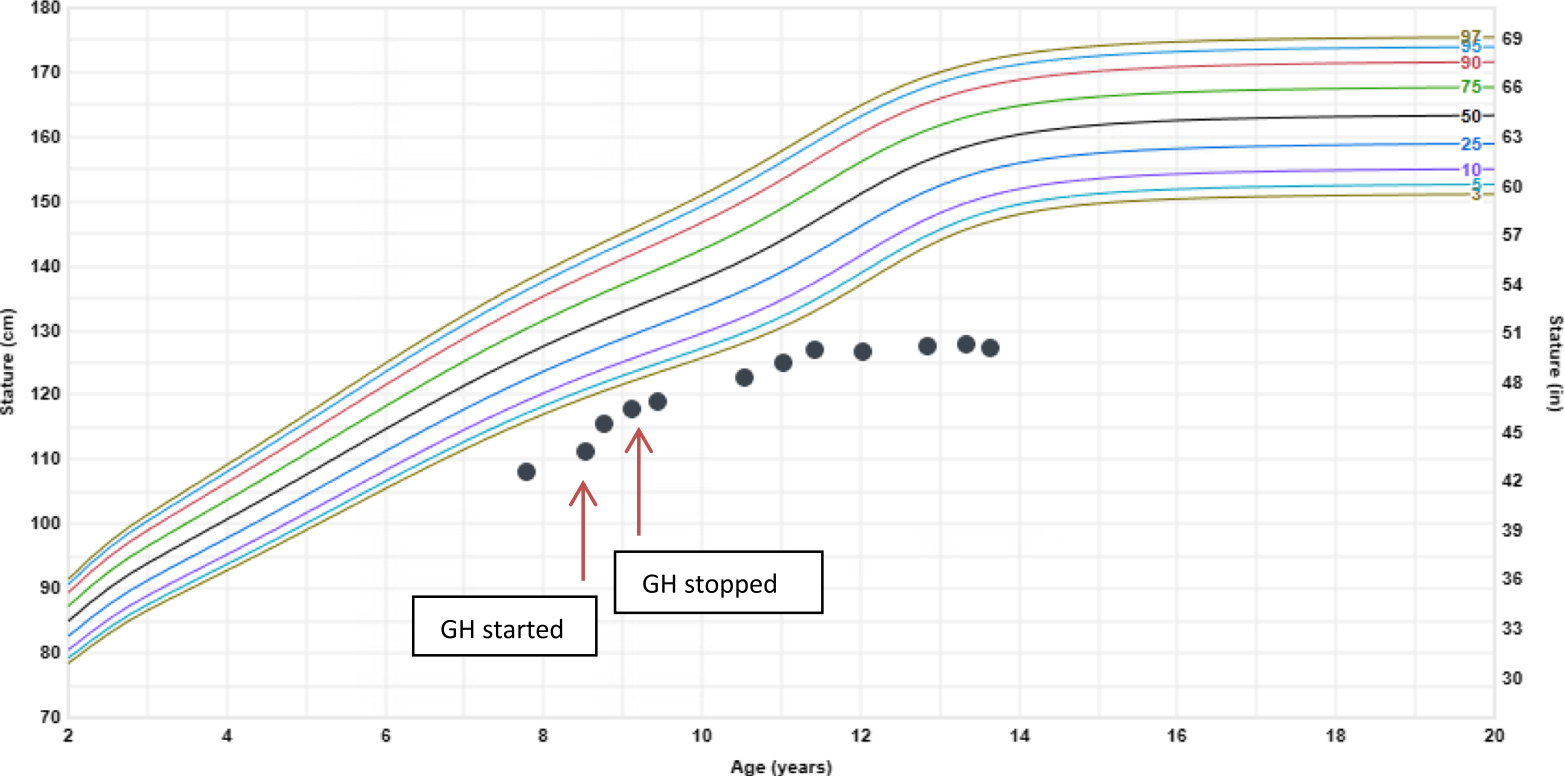
Linear Growth Chart

**Fig. 3:**
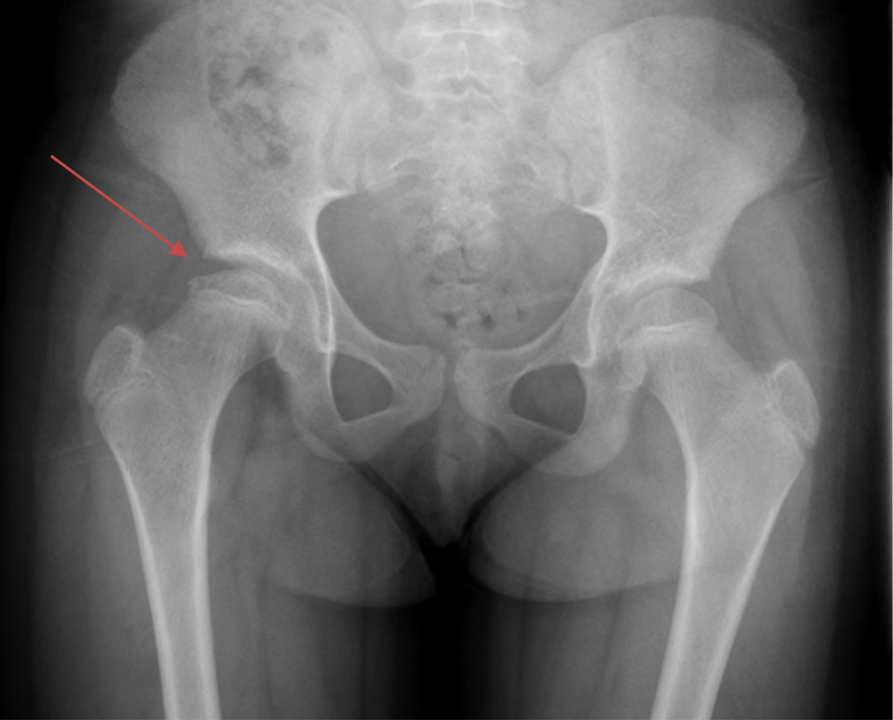
At onset of knee pain: Right capital femoral epiphyseal height loss was observed without evidence of slippage. No significant sclerosis

After 7 months, her annualized growth velocity had increased from 3 cm/year to 11 cm/year and she had grown 6.5 cm (Fig. 2). However, she also reported right knee pain for 1 week. Radiographs (Fig. 3) revealed flattening of the right femoral head consistent with LCPD. She was made non-weight bearing and GH therapy was discontinued.

**Fig. 4:**
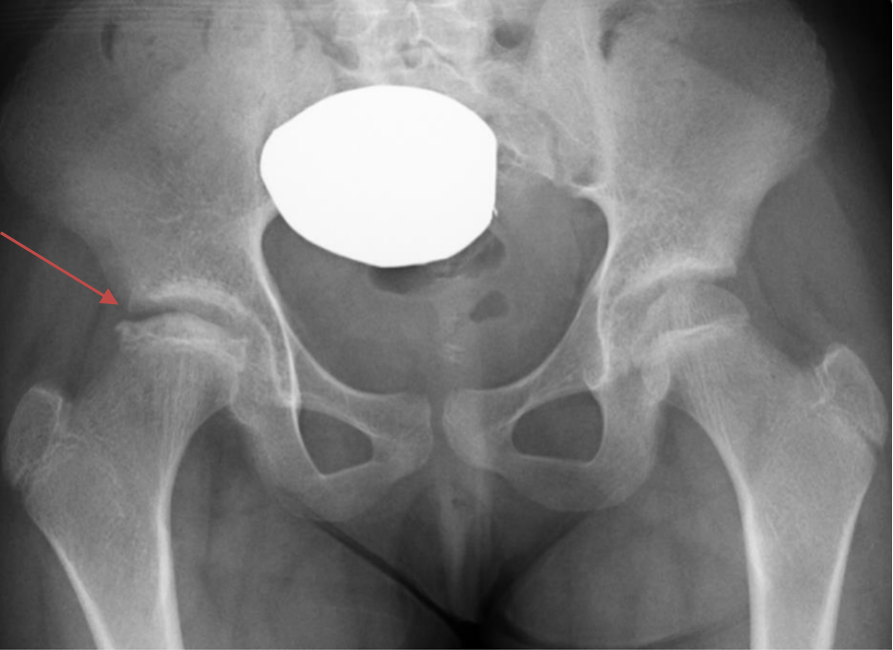
Six weeks later: Increased flattening and sclerosis of right capital femoral epiphysis and mild increased fragmentation laterally

Six weeks later, radiographs (Fig. 4) revealed further collapse with involvement of the entire femoral head, and she was now using crutches/wheelchair. Her lesion stabilized after 8 months with conservative management. She remained off GH, had menarche at 11 years 8 months and reached skeletal maturity at 13 years old with final adult height at 4′2″ (128 cm); Z-score of − 5.5. She is ambulating independently with a leg length discrepancy < 1 cm. She occasionally has right hip pain after walking for long periods and is being monitored closely for early hip arthritis. She has regular menses and continues on levothyroxine and calcitriol for TSH and PTH resistance respectively.

## Discussion

Leg Calves Perthes Disease (LCPD), also called avascular necrosis of the femoral head epiphysis, is most commonly seen amongst children 4–8 years of age. LCPD is grouped together with slipped capital femoral epiphysis (SCFE) and scoliosis as possible adverse skeletal effects of the rapid growth caused by GH therapy in children with GH deficiency [3]. The incidence rate for LCPD in idiopathic growth hormone deficient patients is reported to be 10 per 100,000 compared with 5–7 per 100,000 for the general population. Interestingly, the rate did not vary if the GH deficient children were on growth hormone therapy or not [4]. LCPD is not known to be associated with ACRDYS, but LCPD was reported in a patient with PHP1A not on GH [5]. Children with PHP1A treated with GH have not been reported to have an increased risk of LCPD. Proposed underlying risks of LCPD include glucocorticoid exposure, genetic mutations of COL2A1, coagulation abnormalities, traumatic injury to the blood supply, transient synovitis, second-hand smoke exposure, and venous congestion [6]. Patients with underlying renal failure or kidney transplants who are on GH therapy are also reported to be at higher risk to develop LCPD [4]. Similar to a previous case report of a pre-pubertal female with GH deficiency who developed LCPD [7], we hypothesize that growth spurt due to GH treatment led to an insufficient blood supply to the epiphysis, resulting in LCPD in our patient. It is currently advised to stop GH treatment in cases of LCPD to decrease the severity of the disease and prevent contralateral disease [7]. This is what we did and she did not develop contralateral disease. The 2018 consensus guidelines for PHP1A patients include a recommendation to screen all patients for GH deficiency [8] and then to treat those who are deficient. Current standard starting dose of GH is 0.3 mg/kg/week divided daily and titrated utilizing the IGF-1 level and the linear growth velocity [2]. For PHP1A children born small for gestational age (SGA), GH treatment can be considered, and typically higher doses are used than for GH deficiency [2, 8]. De Zegher et al. reported higher doses of GH at 0.46 mg/kg/week used in patients with SGA without skeletal dysplasia and GH was well tolerated [9]. Our patient who was born SGA was started on higher GH dose of 0.35 mg/kg/week as compared to GH deficient PHP1A patients. It may be that this higher dose contributed to the development of LCPD.

## Conclusion

GH is the standard of care for patients with PHP1A with GH deficiency and has been shown to increase final adult height significantly and there have been no reported cases as of yet of LCPD on GH. Efficacy of GH in improving final adult height in patients with ACRDYS1 is unknown. The risk of LCPD during GH treatment in patients with ACRDYS1 needs to be considered and patients should be counseled accordingly. Clinicians may also consider using a lower initial GH dose such as 0.16–0.24 mg/kg/week used for growth hormone deficiency [10].

## Data Availability

not applicable.

## Abbreviations

ACRDYS 1: Acrodysostosis type 1
ACRDYS 2: Acrodysostosis type 2
AHO: Albright hereditary osteodystrophy
PHP1A: Pseudohypoparathyroidism type 1A
*GNAS*: Gα-stimulatory subunit
*PRKAR1A*: Protein kinase A regulatory subunit 1A
*PDE4D*: Phosphodiestarase type 4D
PKA: Protein kinase A
GH: Growth hormone
PTH: Parathyroid hormone
TSH: Thyroid stimulating hormone
LH: Luteinizing hormone
FSH: Follicle stimulating hormone
GHRH: Growth hormone releasing hormone
LCPD: Legg-Calve-Perthes Disease
SCFE: Slipped Capital Femoral Epiphysis
IGF-1: Insulin like growth factor-1
IGF-BP3: Insulin like growth factor binding protein 3
